# Liver Resection in Patients With Synchronous Colorectal Liver and Lung Metastases

**DOI:** 10.6004/jadpro.2016.7.7.9

**Published:** 2016-11-01

**Authors:** Allison Horner, Kendall Lencioni

**Affiliations:** The University of Texas MD Anderson Cancer Center, Houston, Texas

According to the Centers for Disease Control and Prevention (CDC), colorectal cancer (CRC) is the second leading cause of cancer death in the United States and is the third most common cancer among men and women (CDC, 2016). Approximately 50% to 60% of patients diagnosed with CRC develop distant metastatic disease (Kim et al., 2012). The two most common sites of distant metastatic disease are the liver and the lungs (Mise, Mehran, Aloia, & Vauthey, 2014). Liver metastases occur in approximately 30% of all CRC cases and account for at least two-thirds of CRC deaths (Kopetz et al., 2009).

Prior to 1990, the presence of colorectal liver metastases (CLM) excluded patients from surgical treatment, leading to a median survival (MS) of 12 months. Over the past 2 decades, advancements in surgical techniques and systemic chemotherapy have improved the 5-year survival rate to 58% (Mise et al., 2015). For patients with CLM, the combination of chemotherapy and surgical resection has been reported to be the most effective treatment to improve overall survival (OS; Vauthey et al., 2013; Kopetz et al., 2009). New surgical techniques and approaches have improved the safety of liver surgery and increased the percentage of patients eligible for resection of CLM to about 25% (Vauthey & Kopetz, 2013).

## MANAGING PATIENTS WITH EXTRAHEPATIC DISEASE

Historically, extrahepatic disease in patients with CLM was considered a contraindication to resection due to its association with poor outcomes (Caprizo et al., 2009). The patients were limited to systemic chemotherapy with or without liver-directed therapies (Hwang et al., 2014). Over the past few decades, there have been continued advancements in available therapies for patients with stage IV CRC. Liver metastases in patients with CRC have been approached as regional disease, which has a better prognosis than other distant sites (Elias et al., 2003).

Targeted drugs for metastatic CRC have continued to develop rapidly, and new chemotherapy drugs have changed the landscape of stage IV disease. First-line chemotherapy for metastatic colorectal cancer includes the use of fluorouracil (5-FU) plus oxaliplatin or irinotecan. This combination can be given with or without the antivascular endothelial growth factor (anti-VEGF) agent, bevacizumab (Avastin; Loupakis et al., 2014). Preoperative, or neoadjuvant, chemotherapy has increasingly been used to test tumor biology and aids in the selection of optimal surgical candidates (Vauthey et al., 2013).

The concept of liver resection in patients with extrahepatic disease is increasingly being challenged. Hwang et al. (2014) looked at 3,481 patients with CLM and extrahepatic metastatic disease involving the lungs, lymph nodes, peritoneum, and other sites (ovaries, bone, spleen, adrenal gland, vagina, and pancreas; Table 1). Of these patients, 78% had an R0 resection (complete resection with negative margins). When these patients were compared with a similar group who did not undergo resection, the resection group was found to have an OS at 5 years of 28% vs. 0% of the group excluded from resection. The MS was 31 months compared with 16 to 24 months for the previous group. This study also found that patients with resected lung metastases had the best outcome compared with patients who had other sites of extrahepatic disease (lung MS at 45 months, lymph nodes at 26 months, and peritoneal disease at 29 months). Those with a lower tumor burden were also found to have a better OS (Hwang et al., 2014).

**Table 1:**
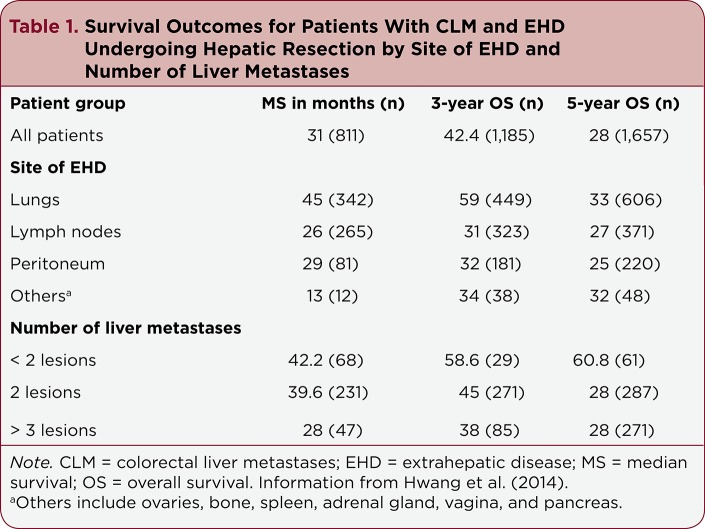
Survival Outcomes for Patients With CLM and EHD Undergoing Hepatic Resection by Site of EHD and Number of Liver Metastases

## SYNCHRONOUS LIVER AND LUNG METASTASES

The presence of resectable lung metastases from CRC is no longer a contraindication for resection of liver metastases (Brouquet et al., 2011). A multidisciplinary approach is critical in managing patients with metastatic CLM and extrahepatic disease. As noted in the Hwang et al. study (2014), the specific site of extrahepatic disease has a direct impact on long-term outcomes.

Since resection of lung metastases in patients with CRC has a more favorable outcome than that of lymph node or peritoneal disease, there has been an increased interest in taking a surgical approach to patients with liver (Table 2) and lung (Table 3) CRC metastases (Maithel et al., 2010; Hwang et al., 2014). Studies have continued to show that assessment of response to chemotherapy, such as a decrease in tumor size on cross-sectional imaging, helps to select those patients with advanced disease who would benefit from an aggressive surgical approach. With improved chemotherapy, novel surgical techniques, and better patient selection, the 5-year OS rate for patients undergoing resection for liver and lung metastases has improved from 21% before the year 2000 to 54% based on recent data (Brouquet et al., 2011; Salah et al., 2014). As patient selection improves, so does the safety of combined liver and lung resections. Overall survival for these patients has subsequently increased.

**Table 2:**
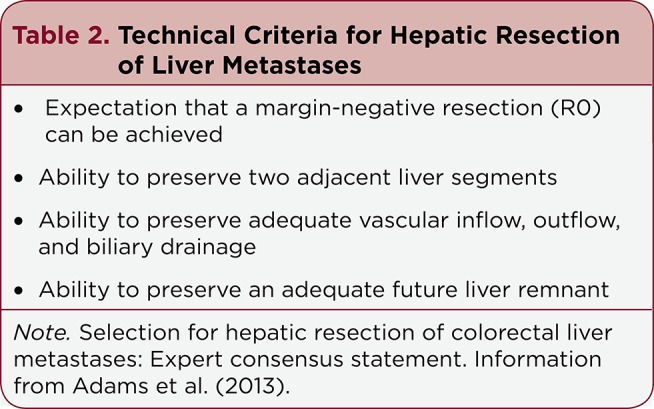
Technical Criteria for Hepatic Resection of Liver Metastases

**Table 3:**
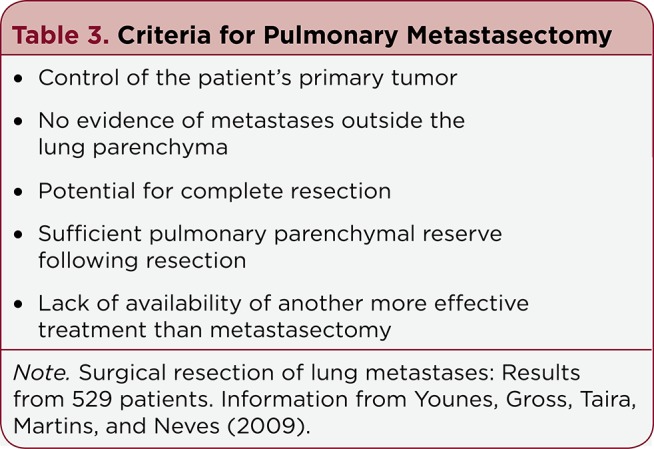
Criteria for Pulmonary Metastasectomy

## RESECTION OF LIVER METASTASES WITHOUT RESECTION OF SYNCHRONOUS LUNG METASTASES

**Figure 1:**
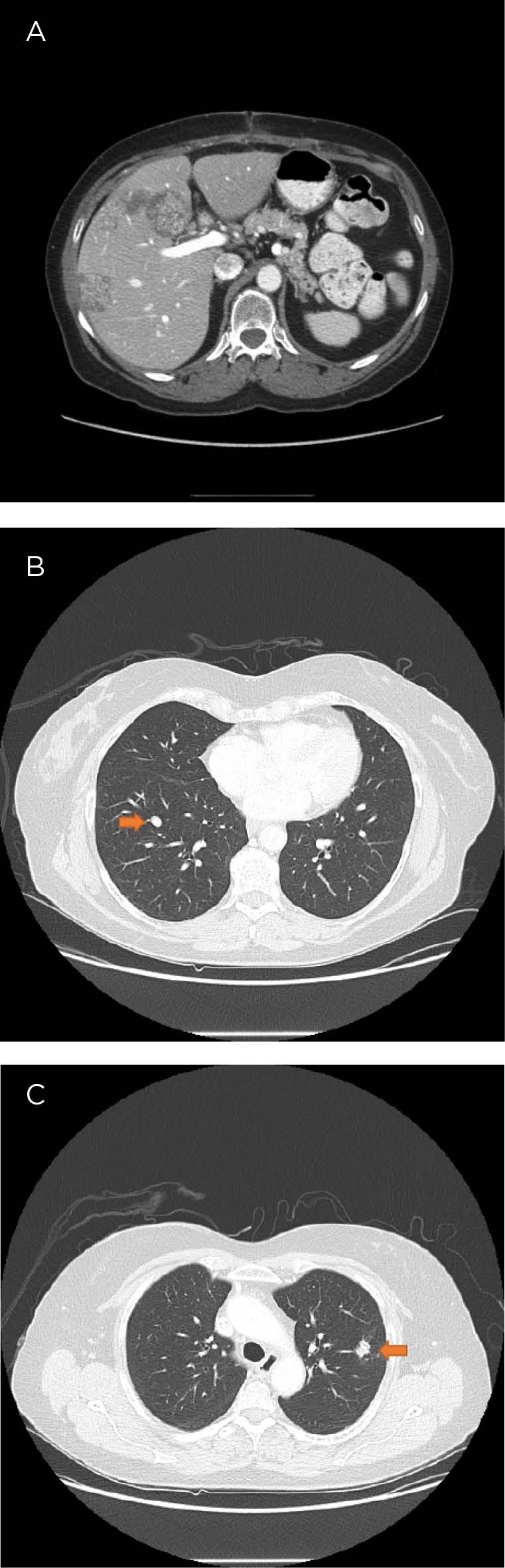
Cross-sectional images of a patient who initially presented with resectable metastases to the liver (A) and lungs, but then disease progressed in the lungs (B, left lung; C, right lung), and the patient did not undergo resection of lung metastases.

Mise et al. (2015) recognized this dilemma and decided to investigate how to refine an approach of rescuing some of these patients for whom liver resection historically has not been indicated. The liver and lungs are the two most common sites of metastases from CRC (Galandiuk et al., 1992). When they are presented synchronously, it is considered advanced disease, and only a few patients are surgical candidates (Headrick et al., 2001; see Figure 1). The most recent data report that liver resection is now performed in about 25% of patients with CLM, with a reported 5-year OS rate of 58% (Kopetz et al., 2009). That leaves an unfortunate rate of 75% of patients with CLM who are not candidates for surgical resection. These patients must then rely on medical treatment alone, which leaves them with a MS of 24 months or less (Hecht et al., 2009). This is quite a troubling dilemma for patients who otherwise have technically resectable liver metastases.

To expand a patient’s candidacy for resection of colorectal liver metastases, the impact of synchronous lung metastases must be clarified. In patients with colorectal lung metastases alone, there has been a doubling in the 30-year OS rate since the 1970s to 35.5% due to improvements in systemic therapy (Mitry et al., 2010). They wanted to test the hypothesis that some patients with unresected lung metastases may still benefit from resection of their CLM between the improvement in patients’ OS with resected CLM alone and in patients with unresectable lung metastases on systemic therapy (Abdalla et al., 2004; Kopetz et al., 2009).

The Mise et al. study (2014) evaluated patients whose lung lesions progressed and were never resected after hepatic metastasectomy. They also reviewed the KRAS status, due to patients with KRAS-mutated tumors having the propensity to have lung metastases and early recurrence in the lungs after resection of CLM (Vauthey et al., 2013). A prospective review of 1,539 patients who underwent resection of CLM from January 2000 to June 2012 was performed. The patient population of synchronous liver and lung metastases included 98 patients who underwent liver resection alone (the study population), 64 patients who received only chemotherapy, and 41 patients who underwent resection of both liver and lung metastases. Preoperative chemotherapy was given to 86% of the study population patients.

Of these patients, lung tumor progression was observed in 19% during chemotherapy. Postoperative chemotherapy was given to 98% of the study population. The median time to progression of lung metastases was 5 months. A total of 60% of patients had progression of lung metastases in 6 months post resection of liver metastases. In 98% of the study population, lung resection was not indicated due to progression of lung metastases, the presence of non-lung metastases, or both lung and non-lung metastases. The remaining 2% of the study population declined further surgery after liver resection.

At the median time of follow-up (29 months), 71% of the patients had died, at a median of 27 months after liver resection. The 3- and 5-year OS rates for patients who underwent resection of the CLM but not the lung metastases were 42.9% and 13.1%, respectively. This is quite an improvement compared with 3- and 5- year OS rates for patients who had chemotherapy alone (no liver or lung resection) at 14.1% and 1.6%, respectively, but expectedly worse than those who had resection of liver and lung metastases at 68.9% and 56.9%, respectively. The OS rate for patients in this study was 34 months compared with those with chemotherapy alone at 17 months and those who had resection of both liver and lung metastases at 64 months. A *KRAS* mutation and rectal primary tumors were associated with poorer outcomes.

Overall, this study revealed an intermediate survival for patients who had resection of CLM in the setting of no resection for the lung metastases. Based on these findings, Mise et al (2014) expressed there may be a role for resection of CLM in patients whose lung metastases are unresectable, especially in light of improved advancements in surgical techniques, systemic therapy, and patient selection.

## DISCUSSION

With advancements in chemotherapy and aggressive approaches in surgery, there has been a significant improvement in OS of patients with resectable CLM, with 5-year survival rates approaching 60% (Abdalla et al., 2004; Kopetz et al., 2009). Historically, the presence of extrahepatic disease has limited treatment options for patients to chemotherapy and other nonsurgical therapies. However, improved cross-sectional imaging, chemotherapy, surgical techniques, and patient selection have led to challenging the idea that extrahepatic disease is a contraindication to liver resection (Elias et al., 2003).

The dilemma we currently face is what to do with patient populations such as the one discussed in this article, where CLM are resectable but lung metastases are not. Mise et al. are among those who want to challenge historic ideas and look for more aggressive treatment strategies. Their findings of intermediate but improved survival in these patients of resected CLM in the setting of unresectable lung metastases have led others to further investigate this possible option. This may lead to more treatment options and possibly improved survival.

The Mise et al. study (2014) briefly touched on how KRAS-mutated vs. wild-type status and rectal vs. colon primary may be associated with poorer outcomes, potentially contributing to improvement in patient selection. Further directions could include looking at carcinoembryonic antigen levels, tumor burden, length and specific types of chemotherapy (preoperative and postoperative), other genetic mutation analyses, and how each of these factors or a combination of these factors impacts overall prognosis and survival. The resection of CLM in the setting of other areas of unresectable extrahepatic disease beyond the lungs could also be investigated for a possible survival benefit. Research is ongoing in the search for additional aggressive approaches to the treatment of metastatic CRC.

**Figure 2:**
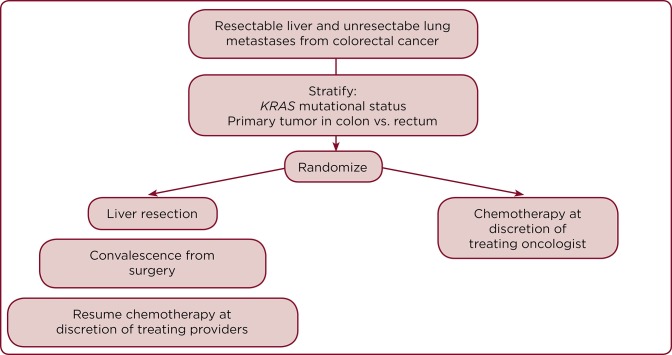
Schema for "Randomized Controlled Phase II Trial of Liver Resection vs. No Surgery in Patients With Liver and Unresectable Pulmonary Metastases From Colorectal Cancer." Reproduced with permission from Dr. Yun Shin Chun.

The positive findings of the Mise et al. study (2014) have prompted interest for a clinical trial to further study their patient population. As recently as May 2016, The University of Texas MD Anderson Cancer Center opened a clinical trial entitled "Randomized Controlled Phase II Trial of Liver Resection vs. No Surgery in Patients With Liver and Unresectable Pulmonary Metastases From Colorectal Cancer." The proposed study is expected to identify a selection of patients most likely to benefit from CLM resection in the setting of unresectable pulmonary metastases (Figure 2).

## IMPLICATIONS FOR ADVANCED PRACTITIONERS

Advanced practitioners in oncology are often the first to evaluate these patients with CRC and thus play a large role in patient selection and in the overall management of these patients in all stages of disease. They are also most often the ones ordering and interpreting tests, assisting in developing treatment plans, and educating patients on the natural disease process and current treatment options. It is important for advanced practitioners to have a thorough understanding of current-day trends in treatment as well as those coming down the pipeline. Having this depth of understanding will assist in patient selection for more aggressive treatment options, such as that proposed by Mise et al. (2014), which could potentially offer some patients a path to improved OS.
